# Both reactive and proactive control are deficient in children with ADHD and predictive of clinical symptoms

**DOI:** 10.1038/s41398-023-02471-w

**Published:** 2023-05-26

**Authors:** Weidong Cai, Stacie L. Warren, Katherine Duberg, Angela Yu, Stephen P. Hinshaw, Vinod Menon

**Affiliations:** 1 Department of Psychiatry & Behavioral Sciences, Stanford University School of Medicine, Stanford, CA USA; 2 Wu Tsai Neuroscience Institute, Stanford University, Stanford, CA USA; 3 Department of Psychology, School of Behavioral and Brain Sciences, The University of Texas at Dallas, Richardson, TX USA; 4 Department of Cognitive Science, University of California, San Diego, CA USA; 5 Department of Psychology, University of California, Berkeley, CA USA; 6 Department of Psychiatry and Behavioral Sciences, University of California, San Francisco, USA; 7 Department of Neurology & Neurological Sciences, Stanford University School of Medicine, Stanford, CA USA

**Keywords:** Human behaviour, ADHD

## Abstract

Cognitive control deficits are a hallmark of attention deficit hyperactivity disorder (ADHD) in children. Theoretical models posit that cognitive control involves reactive and proactive control processes but their distinct roles and inter-relations in ADHD are not known, and the contributions of proactive control remain vastly understudied. Here, we investigate the dynamic dual cognitive control mechanisms associated with both proactive and reactive control in 50 children with ADHD (16F/34M) and 30 typically developing (TD) children (14F/16M) aged 9–12 years across two different cognitive controls tasks using a within-subject design. We found that while TD children were capable of proactively adapting their response strategies, children with ADHD demonstrated significant deficits in implementing proactive control strategies associated with error monitoring and trial history. Children with ADHD also showed weaker reactive control than TD children, and this finding was replicated across tasks. Furthermore, while proactive and reactive control functions were correlated in TD children, such coordination between the cognitive control mechanisms was not present in children with ADHD. Finally, both reactive and proactive control functions were associated with behavioral problems in ADHD, and multi-dimensional features derived from the dynamic dual cognitive control framework predicted inattention and hyperactivity/impulsivity clinical symptoms. Our findings demonstrate that ADHD in children is characterized by deficits in both proactive and reactive control, and suggest that multi-componential cognitive control measures can serve as robust predictors of clinical symptoms.

## Introduction

Attention deficit hyperactivity disorder (ADHD) is a common neurodevelopment disorder with prevalence rates ranging from 5% to 10% of school-aged children worldwide [1, 2]. Strikingly, diagnostic rates of ADHD have doubled in the last two decades in the United States [3], increasing the need and urgency to better understand pathophysiological mechanisms of the disorder. ADHD is primarily characterized by deficits in attention and cognitive control functions [4, 5]. However, conventional behavioral measures such as accuracy and reaction time (RT) do not capture the complete range of component processes associated with cognitive control, nor are they able to effectively distinguish these processes. Moreover, overt behavioral measures typically have weak to moderate effects in differentiating children with ADHD from typically developing (TD) children [6] and have limited associations with core symptoms of the disorder, such as inattention and hyperactivity/impulsivity [7–10]. Recent theories and behavioral models of cognitive control have suggested that both reactive and proactive control processes [11], which are dynamically modulated by task context, error monitoring, and prior expectations, play an important role in cognitive control [12–14]. However, few studies have systematically examined dynamic, reactive, and proactive cognitive control in childhood ADHD. As ADHD is characterized by heterogeneous patterns of both cognitive impairment [15] and symptom profiles, isolating intermediate phenotypes that are superior to current nosology is critical for improving clinical assessments and treatment response. Here we address this gap and characterize multi-componential processes associated with dynamic dual cognitive control (DCC) mechanisms in children with ADHD and their relation to the cardinal clinical symptoms of the disorder.

**Fig. 1:**
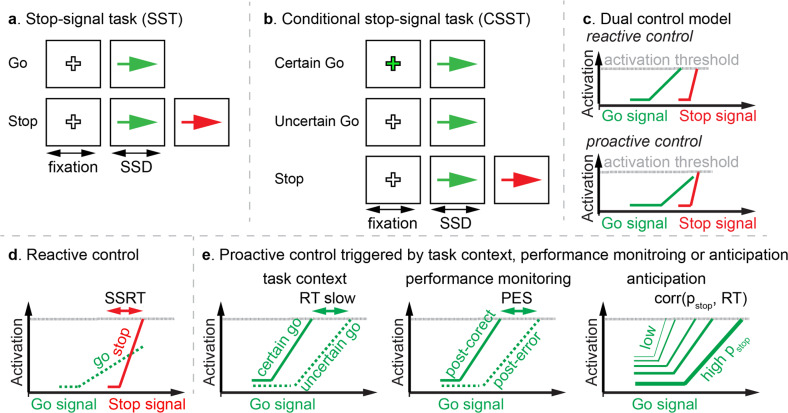
Task paradigms, dual control models, and behavioral measures. **a** Illustration of the stop-signal task (SST). **b** Illustration of the conditional stop-signal task (CSST). **c** Illustration of dual model of cognitive control. **d** Reactive control is measured by the stop-signal reaction time (SSRT), which is estimated based on the Race Model. **e** Proactive control mechanism can be triggered by task context, negative performance feedback, and anticipation of stop signals, which can be measured by RT difference between Uncertain and Certain Go trials in the CSST, post-error slowing in the SST, and correlation between trial-wise anticipation of stop signal and RT in the SST, respectively.

The DCC model posits that there are two distinct operating modes underlying cognitive control: reactive and proactive control [11] (Fig. 1c). Reactive control refers to one’s ability to withhold or override an automatic, habitual, or prepotent process when interference or a countermanding event is detected [11, 16]. For example, a driver who sees a pedestrian suddenly stepping onto the road must quickly inhibit the prepotent action of continuing driving forward instead apply the brake to stop the vehicle. A common behavioral index for reactive control is the stop-signal reaction time (SSRT) in the stop-signal task (SST), which estimates how fast one can cancel a prepotent response [8, 17] (Fig. 1a, d). SSRT is widely used to characterize cognitive control deficits in ADHD [18–20].

Proactive control refers to one’s ability to deploy, in advance, strategies to control an automatic, habitual, or prepotent process given the foreknowledge of interference or a countermanding event [11, 16, 21] (Fig. 1e). For instance, when driving in a busy street, a driver may exercise caution by gently pushing the gas pedal to gradually accelerate the vehicle because the car in the front may slow down or come to a sudden stop and the driver needs to be prepared to respond quickly. Proactive control has primarily been examined using context-driven response strategy adjustments when participants are cued in advance and are indexed in such contexts by longer RTs when a high cognitive load is anticipated [14, 21]. The ability to make adjustments based on performance history is another index of proactive control [22]. Specifically, post-error slowing is a proactive control-related behavioral adaption effect that follows the negative consequence of previous decision-making and is associated with an elongated response time in the following trial [23] (Fig. 1e). However, whether post-error slowing is mainly driven by individual differences in adjustments of response threshold or attention interference remains unresolved [14, 24]. More broadly, proactive control is a dynamic behavioral adaptation process as individuals learn from event history and continuously update their beliefs about the nature of the cognitive task (e.g., the probability of a stop signal in the SST) [12, 25]. Previous studies have found that adult participants proactively adjust their response strategy based on time-varying expectations of task-relevant signals [26, 27] but it is unknown whether children use a similar dynamic proactive control strategy and to what extent such dynamic mechanisms are related to ADHD. Isolating proactive control strategies in children and distinguishing how they impact ADHD has important implications for understanding disorder etiology, mechanistic heterogeneity, developmental trajectories, and treatment response.

Reactive control has been the mainstay of cognitive control studies in ADHD [6, 28, 29]. The most commonly used behavioral measures to quantify reactive control deficits include SSRT in the SST along with errors of commission in continuous performance tasks (e.g., Go/NoGo task) [6, 28, 29]. Both measures have moderate effect sizes in differentiating children with ADHD from TD children [6, 28, 29], with SSRT having a slightly larger effect size than commission errors [6]. Several studies have suggested that SSRT is not a robust predictor of ADHD [18–20] given that many children with ADHD have demonstrated similar SSRTs as those without ADHD [24]. A meta-analysis showed that the effect sizes of SSRT in differentiating children with ADHD from TD children were influenced by task complexity, sex, and comorbidity [30]. However, weak group differences in SSRT reported in early studies may have arisen from small sample sizes (*n* < 20) [31, 32]. Because cognitive control deficits, often operationalized through SST, remain a key component in etiological theories of ADHD [5], it is important to evaluate the reliability of SSRT with different task complexities in children with ADHD with a substantial sample size.

In contrast to reactive control, proactive control has been largely ignored in studies of ADHD despite its potential to uncover robust components of cognitive control dysfunction associated with the disorder. The limited research on proactive control to date has yielded inconsistent findings. One study reported that incarcerated adolescents have more difficulty using proactive control strategies compared to the control group and this difference was associated with a diagnosis of ADHD [33]. Yet another study reported that children with ADHD and TD children have similar proactive control capacities, measured by varying the need for inhibitory control between task runs [34]. Similar findings were reported in a study of boys wherein proactive control was measured by manipulating the probability of stop signals in the SST [35]. Yet the evidence is mixed as other studies have reported less behavioral adaptation, e.g., attenuated post-error slowing, in children with ADHD relative to TD children [36], indicating that at least some children with ADHD have difficulty in proactively adjusting their response strategy after receiving negative feedback. The limited research leaves unclear the extent and sources of proactive control deficits in ADHD. Crucially, to the best of our knowledge, no study to date has examined dynamic DCC mechanisms associated with time-varying demands of proactive and reactive control in children with ADHD. Moreover, their links to clinical symptoms of ADHD are also not known. Here we systematically investigate dynamic DCC mechanisms in children with ADHD, with a focus on proactive control mechanisms under different conditions, and tested their associations with core ADHD symptoms.

To address these critical gaps in the literature, we used two experiments involving a standard SST and a cue-based stop signal task (CSST) [17, 37, 38] to probe dynamic DCC processes in children with ADHD and matched TD children. Reactive control was measured on both the SST and the CSST using standard SSRT estimation procedures [17, 39]. The SST required participants to make an accurate and speedy button-press response corresponding to a left- or right-pointing arrow (Go signal) and to withhold responses when the arrow changed color (Stop signal). SSRT was determined by the distribution of Go RT and response rate in Stop trials based on the Race Model [39]. SSRT is an optimal measure for reactive control and it has a strong association with stopping-related neural activity in cortical–subcortical circuits [40, 41]. SSRT was also estimated in the CSST in the second experiment, using similar procedures as in the SST. Together, SST and CSST allowed us to examine the reliability of SSRT under different task complexity conditions.

Proactive control, including context-, performance-, and anticipation-driven proactive control, were examined separately in the CSST and SST using both model-free and model-based approaches (Fig. 1e). The CSST is designed to probe context-driven proactive control. The task consisted of Certain and Uncertain Go trials. In the Certain Go trials, a color cue was used to indicate that there would be no stop signals. In Uncertain Go trials, a different color cue indicated that a Stop signal may follow a Go signal. The context-driven proactive control was measured by the extent to which responses were slowed in the Uncertain, relative to Certain, Go trials in the CSST.

Next, performance-driven proactive control, or post-error slowing, was measured by differences in RT between Go trials after unsuccessful stop trials (GoPUS) and Go trials after successful Go trials (GoPSG) in the SST (Fig. 1e). To further determine latent cognitive components that may undermine feedback-driven proactive control in children with ADHD, we used a drift-diffusion model (DDM) to estimate three key decision-making components associated with post-error slowing: decision threshold, drift rate, and non-decision time [42, 43].

Last, anticipation-driven proactive control was determined by the extent to which Go RT was modulated by the trial-wise expectation of stop signals in the SST. The dynamic belief model (DBM) [12] was used to estimate trial-wise anticipation of the likelihood of stop signal (p_stop_) and anticipation-driven proactive control capacity was quantified by the correlation between trial-wise Go RT and p_stop_ [12, 26, 27] (Fig. 1e). Here we investigated whether children with ADHD can dynamically and effectively update their belief about the probability of a stop signal from event history and modulate their proactive control efforts accordingly.

Finally, little is known about the relationship between reactive and proactive control in children and to what extent the dual control mechanisms predict core symptoms of ADHD, such as inattention and impulsivity/hyperactivity. Here we purposely examined whether children who have better reactive control also have greater proactive control function and tested whether component measures of reactive and proactive control can predict core symptoms of ADHD.

We hypothesized that children with ADHD would have longer SSRT relative to TD children. We also predicted that children with ADHD would have smaller context-driven response slowing and less post-error slowing than TD children. The DDM allowed us to further determine the contribution of latent decision-making components underlying post-error slowing [44]. We hypothesized that if weak post-error slowing was related to difficulty in the timely adjustment of the decision boundary, children with ADHD would show smaller post-error-related changes in response threshold than TD children. In contrast, if weak post-error slowing was linked to less post-error interference, perhaps arising from poor self-awareness of mistakes, children with ADHD would exhibit smaller post-error-related changes in drift rate than TD children. Moreover, we predicted that children with ADHD would exhibit poorer anticipation-driven proactive control, evidenced by a smaller correlation between trial-wise Go RT and p_stop_, than TD children. We further predicted that TD children who have better reactive control would also have better proactive control but this relation may be dampened in children with ADHD. We also hypothesized that behavioral measures from the dynamic DCC model would predict ADHD clinical symptoms, such as inattention and hyperactivity/impulsivity.

## Results

### Participants’ demographics

One hundred and seven children (9–12 years old) were recruited from the local community. 50 children with ADHD (16F/34M, 11 ± 1 years old) and 30 TD children (14F/16M, 11 ± 1 years old) who completed two runs of SST and two runs of CSST and met task performance criteria (see the “Methods” section for details) were included in the analyses. Table 1 summarizes participants’ demographic information, ADHD symptoms, and behavioral performance in both the SST and CSST.

**Table 1:** Participants’ demographic information and behavioral performance.

	All	TD	ADHD	t/chi-stats	*p*-value
Sample size	80	30	50		
Age (years old)	11 ± 1	11 ± 1	11 ± 1	0.32	0.75
Gender (F/M)	30/50	14/16	16/34	1.15	0.28
Verbal IQ	107 ± 13	109 ± 13	105 ± 13	1.18	0.24
Inattention (Conners)	69 ± 18	48 ± 6	80 ± 11	16.91	2.20E−16
Hyper/Impul (Conners)	66 ± 19	47 ± 7	77 ± 15	12.07	2.20E−16
Inattention (SWAN)	(−6) ± 13	9 ± 9	(−15) ± 6	12.68	2.20E−16
Hyper/Impul (SWAN	(−1) ± 13	11 ± 10	(−9) ± 8	9.62	2.75E−13
*SST*					
Go Accuracy (%)	92 ± 8	94 ± 6	90 ± 9	2.94	0.004
Go RT mean (ms)	528 ± 76	497 ± 61	547 ± 79	3.15	0.002
Go RT std (ms)	127 ± 46	107 ± 38	138 ± 47	3.25	0.002
Stop Accuracy (%)	51 ± 5	51 ± 3	51 ± 6	0.29	0.77
UnsuccStop RT mean (ms)	471 ± 67	446 ± 56	486 ± 68	2.82	0.006
UnsuccStop RT std (ms)	86 ± 49	68 ± 29	97 ± 55	3.09	0.002
SSD (ms)	196 ± 59	191 ± 52	199 ± 64	0.6	0.55
SSRT (ms)	302 ± 54	282 ± 42	314 ± 57	2.85	0.006
GoPUS-GoPSG RT (ms)	24 ± 50	42 ± 44	13 ± 51	2.66	0.01
StopPUS-StopPSG ACC (%)	14 ± 16	20 ± 14	11 ± 17	2.72	0.008
Corr Pstop and Go RT	0.13 ± 0.13	0.19 ± 0.14	0.10 ± 0.10	3.24	0.002
*CSST*					
Uncertain Go Accuracy (%)	91 ± 10	94 ± 8	90 ± 11	1.76	0.08
Uncertain Go RT mean (ms)	539 ± 88	501 ± 56	562 ± 96	3.68	0.0005
Uncertain Go RT std (ms)	131 ± 53	114 ± 38	141 ± 58	2.46	0.02
Stop Accuracy (%)	53 ± 7	52 ± 7	53 ± 7	0.61	0.55
UnsuccStop RT mean (ms)	474 ± 64	443 ± 49	493 ± 63	3.88	0.0002
UnsuccStop RT std (ms)	195 ± 69	66 ± 25	112 ± 81	3.69	0.0004
SSD (ms)	139 ± 51	186 ± 53	191 ± 51	0.44	0.66
SSRT (ms)	310 ± 57	285 ± 51	326 ± 56	3.31	0.001
Certain Go Accuracy (%)	90 ± 10	91 ± 10	89 ± 11	1.01	0.32
Certain Go RT mean (ms)	511 ± 85	470 ± 51	536 ± 92	4.13	9.12E−05
Uncertain Go–Certain Go (ms)	28 ± 33	31 ± 26	26 ± 37	0.65	0.52

Children with ADHD and TD controls did not differ in age, sex, and verbal IQ (all *p*s > 0.2, two-sample two-tailed *t*-test). Children with ADHD had severe inattention and hyperactivity and impulsivity symptoms relative to TD children (*p*s < 0.001, two-sample two-tailed *t*-test, Table 1).

### Overall behavioral performance in the SST

Participants achieved good performance in the SST and with high accuracy and fast RT on Go trials and targeted accuracy (close to 50%) on Stop trials. RT on UnsuccStop trials was significantly shorter than on Go trials (*t*_79_ = 18.96, *p* < 2.2E−16, one sample two-tailed *t*-test), suggesting no violation of the Race Model [17, 39].

### Reactive control in children with ADHD in the SST

**Fig. 2:**
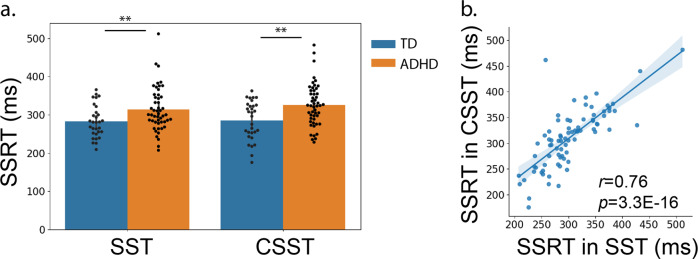
Children with ADHD show generally slow motor control. **a** Children with ADHD have significantly longer RT in Go trials in the SST and Uncertain and Certain Go trials in the CSST than TD children (all *p*s < 0.01). **b** Children with ADHD have significantly longer SSRT in the SST and CSST (all *p*s < 0.01).

We tested whether children with ADHD exhibited a reactive control deficit in the SST. Children with ADHD had worse Go accuracy and longer average RTs and greater RT standard deviations in both Go and UnsuccStop trials than TD children (all *p*s < 0.01, two-sample two-tailed *t*-test, Table 1). No significant group difference was found on Stop accuracy (*p* = 0.77, two-sample two-tailed *t*-test). This pattern indicates that performance-based step-wise adjustments of SSD were implemented successfully in both groups. Children with ADHD had significantly longer SSRT than TD children (*t*_74.48_ = 2.85, *p* = 0.006, *Cohen’s* *d* = 0.61, two-sample two-tailed *t*-test, Fig. 2a). The 95% confidence interval of SSRTs was from 266 to 298 ms in TD children and from 298 to 330 ms in children with ADHD. These findings suggest worse reactive control ability relative to TD children.

### Overall behavioral performance in the CSST

Participants achieved good performance in the CSST with high accuracy and fast RT on Go trials and targeted accuracy (close to 50%) on Stop trials. RT on UnsuccStop trials was significantly shorter than on Uncertain Go trials (*t*_79_ = 13.55, *p* < 2.2E−16, one sample two-tailed *t*-test), suggesting no violation of the Race Model [17, 39].

### Reactive control in children with ADHD in the CSST

We tested whether children with ADHD exhibited a reactive control deficit in the CSST. Children with ADHD had longer average RTs and greater RT standard deviations in both Uncertain Go and UnsuccStop trials than TD children (all *p*s < 0.05, two-sample two-tailed *t*-test, Table 1). Again, we found a significant group difference in SSRT with longer SSRT in children with ADHD than TD children (*t*_65.73_ = 3.31, *p* = 0.001, *Cohen’s* *d* = 0.75, two-sample two-tailed *t*-test, Fig. 2a). The 95% confidence interval of SSRTs was from 266 to 304 ms in TD children and from 310 to 341 ms in children with ADHD.

Next, we examined intra-subject reliability of the SSRT estimation. Indeed, there was a strong correlation between SST and CSST in SSRT (*r*_78_ = 0.76, *p* = 3.3E−16, Pearson’s correlation, Fig. 2b). This finding suggests that the difference in SSRT between children with ADHD and TD children has high test–retest stability across different tasks.

### Proactive control in children with ADHD in the CSST: influence of task context

**Fig. 3:**
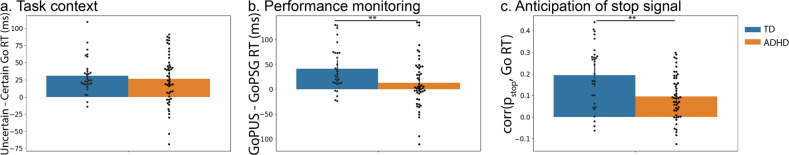
Different proactive control performances in children with ADHD and TD children. **a** Children with ADHD have similar context-dependent proactive control performance as TD children. **b** Children with ADHD have significantly smaller negative feedback-induced response slowing (or post-error slowing) than TD children (*p* < 0.01). **c** Children with ADHD have smaller anticipation-induced response slowing than TD children (*p* < 0.01).

To further investigate whether children with ADHD have difficulty in adjusting their response strategy we examined how task context cues influence response slowing. Proactive control, induced by task context, was measured using the RT difference between Uncertain Go and Certain Go in the CSST. We found that there was a significant context-induced proactive control in children (28 ± 33 ms, *t*_79_ = 7.58, *p* = 5.6e−11, one sample two-tailed *t*-test, Table 1), but there was not a significant group difference between children with ADHD and TD children (*t*_75.85_ = 0.65, *p* = 0.52, two-sample two-tailed *t*-test, Fig. 3a), suggesting that children with ADHD are capable of using prior knowledge to adjust their response strategies.

### Proactive control in children with ADHD in the SST: influence of performance monitoring

We next investigated whether children with ADHD have difficulty adjusting their response strategy based on performance monitoring in the SST. Although no explicit feedback was given in each trial, subjects were fully aware of whether they had made a button press or not as their motor responses served as implicit feedback. This was demonstrated by a significant post-error slowing effect in TD children (42 ± 44 ms, *t*_29_ = 5.17, *p* < 0.001, one sample two-tailed *t*-test, Table 1). Post-error slowing was measured using the difference in RT between Go trials after Unsuccessful Stop trials (GoPUS) and Go trials after Successful Go trials (GoPSG).

We found that children with ADHD had significantly smaller post-error slowing than TD children (*t*_67.99_ = 2.65, *p* = 0.01, *Cohen’s* *d* = 0.60, two-sample two-tailed *t*-test, Fig. 3b). The 95% confidence interval of post-error slowing was from 25 to 58 ms in TD children and from −1 to 27 ms in children with ADHD. This finding suggests that children with ADHD have poorer proactive control ability associated with response slowing triggered by performance monitoring or errors.

To further examine whether post-error slowing benefits stopping performance, we compared the accuracy of Stop trials after Unsuccessful Stop trials (StopPUS) and Stop trials after Successful Go trials (StopPSG) and found a significant post-error effect on stop accuracy (14 ± 16%, *t*_79_ = 8.01, *p* < 0.001, one sample two-tailed *t*-test, Table 1) in all young participants. Moreover, post-error slowing was significantly correlated with the post-error effect on stop accuracy (*r* = 0.23, *p* = 0.04, *Pearson’s* correlation), suggesting that individuals who made more adjustments on reaction time (slowing more) benefitted in stopping accuracy. We also found that TD children showed a greater post-error effect on stop accuracy than children with ADHD (TD: 21 ± 14%, ADHD: 11 ± 17%, *t*_71.14_ = 2.72, *p* = 0.008, *Cohen’s* *d* = 0.60, two-sample two-tailed *t*-test).). The 95% confidence interval of post-error effect on stop accuracy was from 15% to 26% in TD children and from 6% to 16% in children with ADHD.

To better understand why children with ADHD did not slow down their responses after committing wrong responses as much as TD children do, we applied a drift-diffusion model to unveil decision-making components, including threshold, drift rate, and non-decision time, for each GoPUS and GoPSG trial per participant. We then computed differences in each decision-making component between GoPUS and GoPSG, which was further used to test whether performance monitoring-induced changes in decision-making components would differentiate children with ADHD from TD children.

**Fig. 4:**
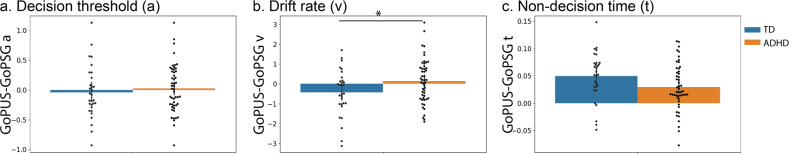
Post-error decision-making processes are altered in children with ADHD. Children with ADHD have similar post-error changes in **a** decision threshold and **b** non-decision time as TD children. **c** Children with ADHD have significantly smaller post-error changes in drift rate than TD children (*p* < 0.05).

We found that performance monitoring-induced changes in response threshold were not significantly different between children with ADHD and TD children (*t*_56.91_ = 0.69, *p* = 0.50, two-sample two-tailed *t*-test, Fig. 4a). Interestingly, negative-feedback induced changes in drift rate were significantly smaller in children with ADHD than TD children (*t*_59.68_ = 2.14, *p* = 0.03, *Cohen’s* *d* = 0.50, two-sample two-tailed *t*-test, Fig. 4b). Negative-feedback induced changes in non-decision time were marginally significantly smaller in children with ADHD than TD children (*t*_59.35_ = 1.98, *p* = 0.05, *Cohen’s* *d* = 0.46, two-sample *t*-test, Fig. 4c). The 95% confidence interval of post-error effect on drift rate was from 3.18 to 3.89 in TD children and from 3.21 to 3.78 in children with ADHD. These findings suggest that errors have less interference on information accumulation speed in children with ADHD than in TD children.

### Proactive control in children with ADHD in the CSST: influence of event history and anticipation

Next, we examined proactive control associated with the anticipation of stop signals. We used DBM to measure trial-wise anticipation of stop signals (p_stop_). We then computed the correlation between p_stop_ and RT across Go trials for each child, with higher correlations indicating more response slowing when participants believe that the stop signal is more likely to occur. We found that children with ADHD had significantly lower correlations than TD children (*t*_49.14_ = 3.12, *p* = 0.002, *Cohen’s* *d* = 0.77, two sample two-tailed *t*-test, Fig. 3c), suggesting that children with ADHD are not as effective as TD children on adjusting their response strategy based on anticipation of stop signals. The 95% confidence interval of the correlation between p_stop_ and Go RT was from 0.13 to 0.23 in TD children and from 0.06 to 0.12 in children with ADHD. Results suggest that children with ADHD have poorer proactive control ability associated with anticipation-related response slowing.

### Reactive and proactive control measures are correlated in TD children but not in children with ADHD

Next, we examined whether reactive control and proactive control functions are correlated in children as previously demonstrated in adults [37].

**Fig. 5:**
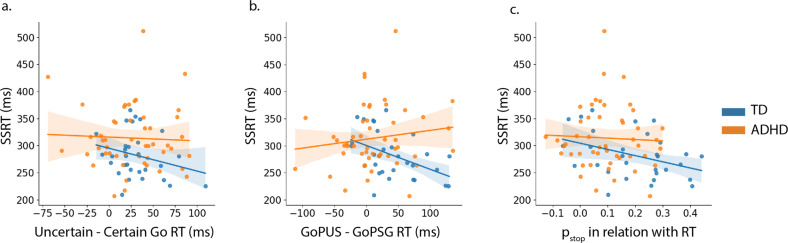
Proactive control is correlated with reactive control in TD children but not in children with ADHD. **a** Context-dependent response slowing is significantly correlated with SSRT in TD children (*r* = −0.45, *p* = 0.01) but not in children with ADHD (*r* = 0.24, *p* = 0.09). **b** Post-error slowing is significantly correlated with SSRT in TD children (*r* = −0.46, *p* = 0.01) but not in children with ADHD (*r* = 0.15, *p* = 0.31). **c** Correlation coefficient between trial-wise stop signal anticipation (p_stop_) and RT is significantly correlated with SSRT in TD children (*r* = −0.38, *p* = 0.04) but not in children with ADHD (*r* = 0.02, *p* = 0.85).

Proactive control induced by task context had no significant correlation with SSRT in the whole cohort (*r*_78_ = −0.11, *p* = 0.31, *Pearson’s* correlation). When examining each group separately, however, we found a significant correlation in TD children (*r*_28_ = −0.45, *p* = 0.01, *Cohen’s d* = 1.01, *Pearson’s* correlation, Fig. 5a) but not in children with ADHD (*r*_48_ = 0.24, *p* = 0.09, *Pearson’s* correlation). A Fisher’s *Z* test confirmed that the correlation coefficients are significantly different between the two groups (*z* = 3.02, *p* = 0.001, *Fisher’s* test).

Proactive control induced by performance monitoring had no significant correlation with SSRT in the whole cohort (*r*_78_ = −0.09, *p* = 0.38, *Pearson’s* correlation). When examining each group separately, however, we found a significant correlation in TD children (*r*_28_ = −0.46, *p* = 0.01, *Cohen’s d* = 1.03, *Pearson’s* correlation, Fig. 5b) but not in children with ADHD (*r*_48_ = 0.15, *p* = 0.31, *Pearson’s* correlation). A Fisher’s *Z* test confirmed that the correlation coefficients are significantly different between the two groups (*z* = 2.69, *p* = 0.004, *Fisher*’s test).

Proactive control induced by anticipation of stop signals had a significant correlation with SSRT in the whole cohort (*r*_78_ = −0.24, *p* = 0.03, *Cohen’s* *d* = 0.49, Pearson’s correlation). When examining each group separately, however, we found a significant correlation in TD children (*r*_28_ = −0.38, *p* = 0.04, *Cohen’s* *d* = 0.82, *Pearson’s* correlation, Fig. 5c) but not in children with ADHD (*r*_48_ = −0.02, *p* = 0.85, *Pearson’s* correlation). A Fisher’s *Z* test demonstrated that the correlation coefficients are marginally significantly different between the two groups (*z* = 1.57, *p* = 0.06, *Fisher’s* test).

Additional multiple linear region analysis using age, gender, and IQ as confounds found that proactive control induced by task context is a significant predictor of SSRT (*p* = 0.02), proactive control induced by performance monitoring is a marginally significant predictor of SSRT (*p* = 0.05), and anticipation of stop signals is a marginally significant predictor of SSRT (*p* = 0.06) in TD children (Supplementary Table S1).

Together, our finding suggests that TD children who have good reactive control also have good proactive control, like adults [37], but this association is not observed in children with ADHD.

### Dual control mechanisms in relation to core symptoms of ADHD

We used the SWAN to examine core symptoms of ADHD in relation to reactive and proactive control ability because it is sensitive to variance in both inattention and hyperactivity/impulsivity dimensions [45].

SSRT in the SST was significantly correlated with Inattention (*r*^2^ = 0.08, *p* = 0.009, *Cohen’s* *d* = 0.60, *Pearson’s* correlation, Supplementary Fig. S1a) and Hyperactivity/Impulsivity scores (*r*^2^ = 0.09, *p* = 0.004, *Cohen’s* *d* = 0.65, *Pearson’s* correlation, Supplementary Fig. S1b), and SSRT in the CSST were also significantly correlated with Inattention (*r*^2^ = 0.13, *p* = 0.001, *Cohen’s d* = 0.77, *Pearson’s* correlation, Supplementary Fig. S1c) and Hyperactivity/Impulsivity scores (*r*^2^ = 0.10, *p* = 0.003, *Cohen’s d* = 0.68, *Pearson’s* correlation, Supplementary Fig. S1d).

Proactive control induced by task context cues was not significantly correlated with Inattention and Hyperactivity/Impulsivity scores (all *p*s > 0.5, *Pearson’s* correlation). Proactive control induced by performance monitoring was significantly correlated with Hyperactivity/Impulsivity scores (*r*^2^ = 0.11, *p* = 0.003, *Cohen’s d* = 0.70, Pearson’s correlation, Supplementary Fig. S2b) and marginally with Inattention (*r*^2^ = 0.04, *p* = 0.06, *Cohen’s d* = 0.41, *Pearson’s* correlation, Supplementary Fig. S2a). Proactive control induced by anticipation of stop signals was significantly correlated with Inattention (*r*^2^ = 0.08, *p* = 0.01, *Cohen’s* *d* = 0.58, *Pearson’s* correlation, Supplementary Fig. S2c) and Hyperactivity/Impulsivity scores (*r*^2^ = 0.10, *p* = 0.004, *Cohen’s d* = 0.65, *Pearson’s* correlation, Supplementary Fig. S2d).

Additional multiple linear region analysis using age, gender, and IQ as confounds confirmed that behavioral measures of dual control mechanisms are a significant predictor of core symptoms of ADHD (Supplementary Table S2).

Together, our findings suggest that dual control functions are associated with both inattention and hyperactivity/impulsivity symptoms.

### Dual control mechanisms predict core symptoms of ADHD

We further examined whether dual control mechanisms can predict core symptoms of ADHD. Specifically, we trained multiple linear regression models based on behavioral measures of reactive and proactive control (i.e., SSRT and context-, performance monitoring-, and anticipation-triggered proactive control) to predict inattention and hyperactivity, and impulsivity scores. A leave-one-out cross-validation procedure was applied, and model performance was assessed by the correlation between predicted and observed clinical scores. We found that behavioral measures indexing dual control mechanisms can significantly predict inattention (*r* = 0.24, *p* = 0.02, *Pearson’s* correlation) and hyperactivity/scores (*r* = 0.36, *p* = 0.001, *Pearson’s* correlation).

Then, we tested whether the inclusion of proactive control measures is important for clinical symptom prediction. To examine this question, we trained the prediction model using only the reactive control measure, SSRT. We found that the predicted and observed inattention scores were marginally significantly correlated (*r* = 0.21, *p* = 0.06, *Pearson’s* correlation) and that the predicted and observed hyperactivity/impulsivity scores were significantly correlated (*r* = 0.24, *p* = 0.02, *Pearson’s* correlation). Furthermore, we determined that prediction models built on both reactive and proactive control measures are marginally more robust than prediction models built on reactive control measures alone in predicting hyperactivity/impulsivity scores (*p* = 0.09, *Pearson and Filon’s* test).

## Discussion

ADHD is a heterogeneous disorder with diverse clinical presentations, cognitive impairments, and symptom trajectories. Although cognitive control deficits are a defining feature of ADHD symptom presentation, the specific component mechanisms remain underspecified and underexplored in understanding etiological contributions and phenotypic presentations. We systematically investigated dynamic DCC mechanisms associated with reactive and proactive control in children with ADHD and their relation to clinical symptoms of ADHD. Going beyond previous studies, we used two different experiments, SST and CSST, to quantify reactive and proactive control using both model-free and model-based approaches. Crucially, our analytic approach allowed us to investigate the influence of task context, performance monitoring, and anticipation of stop signals on the implementation of cognitive control in children with ADHD. We found that relative to TD children, children with ADHD displayed longer SSRT, indicating a weaker reactive control function. For proactive control, children with ADHD demonstrated suboptimal response strategy modulation driven by performance monitoring and the anticipation of stop signals relative to TD children.

Furthermore, in contrast to findings in TD children, reactive and proactive control were not correlated in children with ADHD. These findings suggest that children with ADHD have weaker and less coordinated reactive and proactive control abilities than TD children. Finally, reactive and proactive control weaknesses predicted core symptoms of childhood ADHD, suggesting that the relationship between symptom presentation and performance on behavioral inhibition tasks is more complex than what one reaction time measure can capture. Taken together, our findings highlight specific proactive and reactive cognitive control mechanisms and their disrupted dynamics that are associated with ADHD symptom presentation, advancing our understanding of diverse cognitive profiles.

### Reactive inhibitory control deficits in ADHD are replicable

Prominent cognitive theories of ADHD have argued that deficits in inhibitory control are a cardinal feature of behavioral problems in affected individuals [4]. As such, understanding cognitive components associated with inhibitory control deficits is a major endeavor in ADHD research. Many studies have used response inhibition tasks, such as SST, to probe inhibitory control, and the SSRT is typically conceptualized as reactive (inhibitory) control [11, 16]. Here we used the SST and CSST to probe the reactive inhibitory control function. Averaged SSRTs in children (9–12 years old) were 302 ± 54 ms in the SST and 310 ± 57 ms in the CSST, consistent with SSRTs reported in previous studies using the same age group [46–48]. SSRTs from the SST and CSST were highly correlated, suggesting high intra-individual reliability of SSRTs across cognitive tasks in children. We found that children with ADHD have longer SSRT than TD children. Critically, this finding was replicated in the CSST. Our results are consistent with previous meta-analyses [6, 28, 29, 49] and suggest that relative to TD children, children with ADHD have greater difficulty with and take longer when controlling context-inappropriate impulsive actions. This finding demonstrates that children with ADHD have poor reactive inhibitory control ability relative to TD children.

### Context-driven proactive control is not compromised in ADHD

Proactive control is an important and understudied aspect of cognitive control function in ADHD. The DCC model [11] suggests that proactive control plays an important role in adaptive task-context-based adjustments to response strategy, although this cognitive control component has rarely been tested in childhood ADHD. Proactive control is a common behavioral strategy used to resolve potential future conflicts [11]. Task context cues that indicate an increased possibility of cognitive control demands (e.g., stop signals) are often followed by longer reaction time regardless of whether additional cognitive control is actually needed for a specific trial (e.g., go trial) [14]. Proactive control that is driven by contextual cues has been shown to be accompanied by suppression of excitability in the motor system [21]. One study has suggested that proactive control may recruit similar cortical–subcortical systems as reactive control [38].

In the present study, we used the CSST to probe context-driven proactive control in children. We found that there was significant response slowing in Uncertain Go, relative to Certain Go, trials, confirming that 9–12-year-old children can successfully use task contextual cues to adjust their response strategies [50]. Contrary to our hypothesis, we did not find significant differences in context-driven proactive control between children with ADHD and TD children. Three previous studies have examined proactive control in ADHD [24, 34, 51]. These studies have reported similar findings. A weakness of previous studies is that proactive control was tested in different experimental blocks so that participants knew whether stop signals might occur or not from the very beginning of each task block [24, 34, 51]. In contrast, in the present study, Uncertain Go trials that required proactive control, and Certain Go trials that did not require proactive control, were randomly intermixed within each test administration so that participants needed to dynamically adjust their response strategies based on task contextual cues presented at the beginning of each trial. Our experimental design overcomes the weaknesses of prior studies and provides strong evidence that children with ADHD have preserved the ability in maintaining task-set rules and implementing a context-appropriate response strategy when explicit task cues are provided.

### Proactive control deficits associated with performance monitoring in ADHD

Post-error slowing is one of the most robust measures of adaptive response control [36]. Dominant theories of cognitive control attribute post-error slowing to the implementation of cautious response strategies after an incorrect outcome [52, 53]. A recent study found that corticospinal excitability of the motor cortex is dampened after incorrect responses, suggesting that proactive inhibitory control influences subsequent decision-making [54]. Other studies have argued that an attentional shift or lapse in sustained attention can account for, at least partially, a post-error slowing effect [55]. In the present study, we assessed post-error slowing using RT differences between Go trials after an Unsuccessful Stop trial (the subject mistakenly pressed a button when a stop signal occurred) and Go trials after a Successful Go trial. Although negative feedback was not explicitly given after each trial in this study, participants were aware of the mistakes made as there was a significant post-error slowing effect in children (24 ± 50 ms, Table 1). Consistent with prior work [56], we found that children with ADHD had significantly less post-error slowing than TD children. Although diminished poster-error slowing is often attributed to weaker response caution, a formal process model is needed to isolate the specific cognitive processes that are responsible for the effects demonstrated here. Specifically, diminished post-error slowing in ADHD could also be attributed to distraction of attention, and/or disrupted perceptual and motor processes.

To disentangle the latent cognitive components that contribute to weak post-error slowing in children with ADHD, we used DDM [44]. DDM quantifies latent decision-making processes by estimating three latent components underlying the time to respond on each trial: (1) decision threshold, which indexes the distance to a decision boundary, (2) drift rate, which indexes how fast evidence is accumulated to reach a decision, and (3) a non-decision time, which indexes perception time prior to decision-making and motor response time after decision-making [42, 43]. We specifically modeled decision-making processes underlying Go trials after Unsuccessful Stop trials and Go trials after Successful Go trials, computing the difference to index performance monitoring induced changes in each latent decision-making component. We hypothesized that if weak post-error slowing was due to an impaired or suboptimal ability to adjust their response boundary (i.e., response caution), children with ADHD would show smaller performance monitoring-induced changes in decision threshold than TD children. If children with ADHD paid less attention to errors, they would show smaller performance monitoring-induced changes in drift rate than TD children. Finally, if weak post-error slowing was due to difficulty in adjusting perceptual and motor processes after perceiving the error, children with ADHD would show smaller performance monitoring-induced changes in non-decision time than TD children.

DDM analyses revealed a significant between-group difference in drift rate: children with ADHD demonstrated smaller, performance-monitoring-induced changes than TD children, but not in decision threshold. Our findings support an attentional account, rather than an impaired response caution account, for a weaker post-error slowing effect in children with ADHD. One possible mechanism underlying greater post-error slowing is that children with ADHD may be less aware of errors, leading to a small interference effect on the evidence accumulation. The net effect is that they do not sufficiently alter their decision-making following errors. Interestingly, our previous neuroimaging study showed that children with ADHD have significantly weaker error-related activation in the salience network than TD children [57]. The salience network is important for identifying behaviorally relevant stimuli and is particularly sensitive to errors [58]. Weak engagement of the salience network in response to errors suggests that relative to TD children, children with ADHD may be less sensitive to detecting the saliency of their errors. Our study revealed smaller, performance-monitoring-induced changes in drift rate in children with ADHD, consistent with prior reports of salience network dysfunction in error processing.

### Proactive control deficits associated with anticipation in ADHD

We not only rely on external cues to adjust our response strategies but also make predictions about future events to maximize the benefit-to-cost ratio [12]. In the SST, although the overall probability of stop signals across all trials is pre-defined, the local probability of stop signals varies from trial to trial and individuals adjust their response strategies based on their belief regarding how likely a stop signal will occur in the incoming trial. We used the DBM to estimate individuals’ trial-wise anticipation of the likelihood of a stop signal in the SST based on event history [12, 59]. Previous studies have demonstrated that adult participants implement a more cautious response strategy when they anticipate a high likelihood of stop signals in the coming trials [26, 27]. In the present study, we found that trial-wise anticipation of stop signals was significantly correlated with RT. This finding suggests that, like adults [26, 27], children ages 9–12 years are capable of learning from trial history, updating their belief of incoming signals, and efficiently adjusting their response strategy accordingly. Crucially, this relation was stronger in TD children compared to children with ADHD, suggesting that learning-based behavioral adaptation is less developed in children with ADHD. Children with ADHD may be less effective in updating their belief about the local probability of stop signals arising from a poor ability to track event history. Furthermore, children with ADHD may be less motivated to make effortful, trial-wise response strategy adjustments. Several theories have proposed that ADHD is associated with altered sensitivity to reinforcement, with behavioral and neuroimaging work suggesting that motivational disturbances represent a distinct subcomponent of ADHD [60]. Although we cannot rule out motivational influences, it is likely that deficits in decision-making strategies emerge from the interactive effects of disrupted learning mechanisms such as belief updating and alterations in feedback sensitivity.

### Proactive control in relation to reactive control in children: disrupted dynamics in ADHD

The question of whether proactive control processes influence reactive control has not been investigated in the context of childhood ADHD. Addressing this question has the potential to inform the integrity of dynamic DCC mechanisms underlying cognitive control in ADHD. In neurotypical adults, one previous study has suggested that better proactive control function is correlated with greater reactive control [37]. Here we tested this hypothesis in children and extended prior findings by examining proactive control triggered under different conditions. We found that SSRT was significantly correlated with proactive control in TD children, suggesting that a close relationship between reactive and proactive control exists in children. Beyond the previous finding [37], we further demonstrated that reactive control is not only correlated with context-driven proactive control but also with performance monitoring-driven and anticipation-driven proactive control. More importantly, we determined that in children with ADHD, reactive and proactive control performances were not significantly correlated for all forms of proactive control. One previous study examined post-error slowing in relation to SSRT in children with ADHD and TD children and found no significant relationship between reactive and feedback-driven proactive control [36]. However, clinical and control groups were not evaluated separately. In short, unlike TD children, children with ADHD do not efficiently use reactive and proactive control in conjunction with each to facilitate task performance.

### Disruptions in proactive and reactive control predict core symptoms of ADHD

Prominent cognitive models of ADHD suggest that deficits in inhibitory control underlie behavioral problems in children with ADHD [4, 5]. Although many studies have highlighted deficits in inhibitory control as a cognitive phenotype of the disorder, these studies have predominantly been based on measures of reactive control, and the differential contribution of proactive and reactive control is poorly understood. Furthermore, meta-analytic studies have pointed out that SSRT measures have medium effect sizes in differentiating children with ADHD from TD children [6]. We used multiple model-free and model-based measures from the SST and CSST, including different forms of proactive control induced by task context, performance monitoring, and anticipation, to predict behavioral problems associated with ADHD. These multi-dimensional behavioral features characterize different components of dynamic DCC mechanisms as discussed above.

Our analysis revealed three new results. First, we found a significant relation between multiple measures of reactive and proactive control functions and inattention and hyperactivity/impulsivity scores in children. Importantly, behavioral measures of reactive and proactive control remained robust predictors of clinical symptoms even when adjusting for potential confounds, such as age, sex, and verbal IQ. Second, we trained multiple linear regression models and used a cross-validation procedure to demonstrate that behavioral measures of dynamic DCC mechanisms can predict clinical scores of ADHD in the unseen data. Third, we found that behavioral measures from the dynamic DCC model have better predictive utility of clinical symptoms than behavioral measures from reactive control alone. These findings demonstrate that the DCC model is a robust cognitive framework for uncovering latent cognitive deficits underlying behavioral problems in ADHD.

### Clinical implications

Both proactive and reactive control are important skills for children to develop. For example, children who have difficulty in paying attention in the classroom can use proactive strategies to better control themselves and resist potential distractors, e.g. turning off personal electronic devices. However, when an unexpected situation arises, reactive control is important to help children to inhibit inappropriate behavior and make the more appropriate choice. Problematic cognitive control is fundamental to several etiological theories of ADHD, yet no reliable cognitive profiles have emerged. As children and adolescents with ADHD are at an increased risk for a variety of poor health and social outcomes, identifying clinically meaningful intermediate phenotypes, their neurobiological correlates and pathophysiology, and developmental trajectories is essential to improve prevention and intervention efforts. To render the heterogeneity problem in ADHD more tractable, comprehensive approaches that go beyond reactive control are needed. Here, we synthesized cognitive control task-based performance into proactive and reactive control components, demonstrating that children with ADHD have weaknesses in both and that, unlike the case for their TD peers, proactive and reactive control components were not significantly correlated. Further, dual cognitive control measures demonstrated the better predictive utility of core ADHD symptoms than reactive control measures alone. Importantly, what might appear as a reactive control problem, may be the result of dysfunctional proactive and reactive control dynamics. Such distinctions and precision may be extremely valuable in developing a better nosology for ADHD, as well as improving clinical treatments and prediction. For instance, investigating dual control mechanisms can help determine deficits in proactive and/or reactive control domains, which can allow for further examination of specific treatment responsiveness. This knowledge can help clinicians and researchers develop more targeted and effective treatments for children with ADHD. Finally, our results broadly suggest that children with ADHD may have altered implicit learning as ineffective belief updating and information accumulation speed were observed during conditions in which making errors could have occurred (anticipation) or did occur (performance monitoring).

## Conclusion

We conducted a systematic investigation of cognitive control deficits in children with ADHD in a dynamic framework of a dual cognitive control model. Our findings suggest that relative to their TD peers, children with ADHD suffer from weak reactive control functions. They also have a diminished capacity to learn from trial history and performance and adjust their behavioral strategy accordingly, highlighting deficits in reactive control. The dual cognitive control model is a robust cognitive framework for predicting behavioral problems in ADHD. Our findings thus provide novel insights into understanding the dynamic and multi-componential mechanisms underlying cognitive control deficits in children with ADHD.

## Methods

### Participants

One hundred and seven children (9–12 years old) were recruited from the local community. Informed written consent was obtained from legal guardians of the children and all the study protocols were approved by the Institutional Review Board of Stanford University. Participants who completed two runs of each of SST and CCST and met task performance criteria (Go accuracy is above 50% and Stop accuracy is between 25% and 75%) were included in the final analysis, resulting in 50 children with ADHD (16F/34M, 11 ± 1 years old) and 30 TD children (14F/16M, 11 ± 1 years old). See Table 1 for participant demographic information. The sample size was chosen to maintain a predicted power of 0.8 with a significance level of 0.05 using the effect size of the SSRT difference between children with ADHD and TD children, which is reported in a previous meta-analysis study [6]. Participants who do not meet task performance criteria were not different with respect to clinical symptoms from those included in the data analysis (see Supplementary Results for details).

### Clinical and neuropsychological assessments

Children and their guardians completed a clinical and neuropsychological assessment session. ADHD diagnosis was informed by the children’s guardians and further confirmed using the *Conners* 3rd Edition. ADHD with conduct disorder and oppositional defiant disorder were not excluded as they are common comorbidities [61, 62] (see Supplementary Methods and Results for details). Additional inclusion criterion for both children with ADHD and TD children were the following: no history of claustrophobia, head injury, serious neurological or medical illness, autism, psychosis, mania/bipolar, major depression, learning disability, substance abuse, sensory impairment such as vision or hearing loss, birth weight <2000 g and/or gestational ages of <34 weeks. All children were right-handed with an IQ >80. For all children, inattention and hyperactivity/impulsivity symptoms were assessed using the *Conners* 3rd Edition and Strengths and Weaknesses of Attention-Deficit/Hyperactivity-symptoms and Normal-behavior (SWAN) rating scale. We used the SWAN to investigate the relation between cognitive task performance and clinical symptoms because the SWAN can capture variance between average behavior and far above average range, which is well suited for dimensional analyses [45, 63]. Participants who were under stimulant treatment had gone through a washout period of at least 5 half-lives of the medicine before testing. Details of the medication status of ADHD participants can be found in Supplementary Methods.

### Inhibitory control tasks

#### SST

In the stop-signal task (SST), each trial started with a white cross in the center of the screen for 200 ms and was followed by a green arrow. Participants were told to make a left or right button press response if a left- or right-pointing green arrow (Go signal) was presented, correspondingly. Occasionally (33% chance), the green arrow quickly turned to red (Stop signal) and participants needed to withhold button press responses when the color change was detected. The interval between the onsets of the Go and Stop signals was the stop-signal delay (SSD). The SSD was initiated at 200 ms and its value was adapted based on trial-by-trial performance in a staircase fashion. The SSD increased by 50 ms if a participant successfully withheld a response in the last stop trial; and the SSD decreased by 50 ms if a participant failed withholding a response in the last stop trial. When there was no Stop signal, the Go signal was presented for 500 ms and the response window was 1.5 s. Participants completed two runs of the SST in the scanner and each run included 96 trials (64 Go trials and 32 Stop trials) with jittered inter-trial intervals between 1 and 4 s.

#### CSST

In the cued stop-signal task (CSST), each trial started with a white or green cross (Cue) in the center of the screen for 200 ms and followed by a green arrow. The white cross represented the Uncertain Go trial, such that the green arrow could change to red (33% chance) and participants would need to withhold their response when the green arrow turned to red. In the trials with the white cross, all the parameters were the same as the SST. The green cross represented the Certain Go trial, wherein the green arrow never changed color so no response withholding was needed. Therefore, the white and green crosses represented two different task rules: one with the possibility to stop and the other with no stopping requirement at all. Participants completed two runs of the CSST in the scanner and each run included 80 trials (32 Certain Go, 32 Uncertain Go, and 16 Stop trials) with jittered inter-trial intervals between 1 and 4 s.

### Behavioral measures

*Reactive control* is measured by SSRT in the SST and CSST. First, we examined whether the behavioral data violated a main assumption of the Race Model, that the mean RT in UnsuccStop trials should be shorter than the mean RT in Go trials [39]. Then, SSRT was computed using the integration method based on the Race model [39]: SSRT = *T*−mean SSD, where *T* is the point when the integral of the observed distribution of Go RT in the SST or Uncertain Go RT in the CSST equals the probability of unsuccessful stopping. For each individual, we computed SSRT in the SST and CSST and evaluated the intra-subject reliability of SSRT estimation using *Pearson’s* correlation.

*Context-driven proactive control* is quantified by response slowing modulated by task cues in the CSST, which is Uncertain Go RT relative to Certain Go RT.

*Feedback-driven proactive control* is measured using post-error slowing in the SST, which is the RT difference between GoPUS and GoPSG. Next, DDM was used to further investigate the decision-making processes underlying post-error slow (see below).

*Anticipation-driven proactive control* is measured by the correlation between Go RT and p_stop_ in the SST.

Shapiro-Wilk test was used to test the normal distribution of data. Levene’s test was used to confirm similar variance in behavioral measures between groups (*p*s > 0.05)

### Drift diffusion model: model and parameters

The DDM has been extensively used to estimate two-choice decision-making processes [44]. In this framework, decisions are modeled as a combination of three parameters: threshold (*a*) describing the distance between two decision boundaries, drift rate (*v*) describing the rate at which evidence is accumulated for a given decision, and non-decision time (*t*) which is representative of those aspects of response time not included in decision making (e.g., stimulus encoding, movement execution). Here we used the DDM to disentangle latent decision-making processes underlying post-error slowing. Specifically, the DDM was applied on GoPUS and GoPSG trials to estimate condition-specific decision boundary, drift rate, and non-decision time for each subject. Changes in decision boundary, drift rate, and non-decision time induced by errors were calculated by the differences between GoPUS and GoPSG conditions. Then, between-group differences were tested using two-sample *t*-tests. DDM estimation was conducted using *fast-dm* [43].

### Drift diffusion model: model diagnosis

We carried out model diagnosis analyses to evaluate the goodness-of-fit of the DDM with the behavioral data. The model diagnosis analyses indicated a good model fit of the DDM in the post-error slowing effect. See Supplementary Method and Supplementary Results for details.

### Dynamic belief model: model and parameters

We used a well-validated DBM [12, 27] to estimate trial-wise anticipation of stop signals in the SST for each participant. Here we provide a brief introduction to the DBM. More detailed information and its validation can be found in previous studies [12, 27].

The DBM estimates the belief about the chance of an inhibitory cue occurring in the coming trial based on trial history [59]. On an incoming trial *k*, subjects believe that the chance that an inhibitory cue will occur (Stop trial) is *r*_*k*_ and the chance that no inhibitory cue will occur is 1−*r*_*k*_. The model assumes that subjects believe that *r*_*k*_ has a probability *α* of being the same as *r*_*k*−1_ (the chance that an inhibitory cue occurs in the previous trial) and a probability 1−*α* of being re-sampled from the prior distribution *π*(*r*_*k*_):

$$p\left( {{{{r}}}_{{{k}}}|{{{s}}}_{{{{k}}} - 1}} \right) = \alpha \ast p\left( {{{{r}}}_{{{{k}}} - 1}|{{{s}}}_{{{{k}}} - 1}} \right) + \left( {1 - \alpha } \right) \ast \pi \left( {{{{r}}}_{{{k}}}} \right)$$

where *s*_*k*_ refers to the true trial type of trial *k* (*s*_*k*_ = 1 for Stop trial, *s*_*k*_ = 0 for Go trial); *p*(*r*_*k*−1_|*s*_*k*−1_) refers to the posterior distribution conditional on the last observed trial *k*−1; *π*(*r*_*k*_) is assumed to be a *β* distribution with prior mean pm and shape parameter *scale*.

The model also assumes that subjects update their prior belief using Bayesian inference, and therefore the posterior distribution is computed based on Bayes’ rule:

$$p\left( {{{{r}}}_{{{k}}}|{{{s}}}_{{{k}}}} \right) \propto p\left( {{{{s}}}_{{{k}}}|{{{r}}}_{{{k}}}} \right) \ast p\left( {{{{r}}}_{{{k}}}|{{{s}}}_{{{{k}}} - 1}} \right)$$

The probability of trial *k* being a Stop trial is determined by

$$P\left( {{{{s}}}_{{{k}}} = 1|{{{s}}}_{{{{k}}} - 1}} \right) = {\int} {P\left( {{{{s}}}_{{{k}}} = 1|{{{r}}}_{{{k}}}} \right) \ast p\left( {{{{r}}}_{{{k}}}|{{{s}}}_{{{{k}}} - 1}} \right){{{dr}}}_{{{\mathrm{k}}}}} = {\int} {{{{r}}}_{{{k}}} \ast p\left( {{{{r}}}_{{{k}}}|{{{s}}}_{{{{k}}} - 1}} \right){{{dr}}}_{{{k}}} = \left( {{{{r}}}_{{{k}}}|{{{s}}}_{{{{k}}} - 1}} \right)}$$

In sum, the model allows us to estimate trial-by-trial anticipation of inhibitory cues p_stop_ based on subjects’ trial history (Go or Stop trials). We used the same model parameters {pm and scale}, which define the *β* distribution, as in the previous study since they have been well validated in the stop-signal task [27]. We optimized the model parameter *α*, which defines the re-sampling rate from the prior distribution, using an independent dataset (see below).

### Dynamic belief model: optimizing model parameters

Because the parameters in the DBM were tuned based on behavioral data from adult participants [12, 27], we optimized the model parameters to better fit performance in children. Specifically, we used an independent dataset involving 38 children (9–12 years old, 12F/26M, no history of neurological and psychiatric disorders, Supplementary Methods) [46]. Legal guardians of the young participants provided written consent. Each child completed two runs of the same stop signal task, including 96 trials per run.

To find out the optimal α for the young participants, we gradually changed *α* from 0.2 to 0.7 with increments of 0.05 and determine the saturation value of *α* by how well the model fits the data. To test model fitting, we examined the correlation between go RT and p_stop_ on aggregate trial-wise data across participants. The assumption is that participants will adjust their response strategy (i.e., be more cautious in making responses or wait for the stop signal) when they have high expectations for the occurrence of a stop signal in the coming trial. Specifically, for each *α*, we binned the data for each small range of p_stop_, computed averaged p_stop_ and go RT within each bin, and calculated the correlation between binned p_stop_ and go RT. The optimal α for the young participant is 0.3. Details of this analysis can be found in the Supplementary Results and Supplementary Figs. S3 and S4.

To further examine the robustness of our finding with respect to the choice of the model parameter, we repeated the same analyses with *α* varying from 0.2 to 0.7 and replicated all the main findings (see details in Supplementary Results and Supplementary Table S3).

### Dynamic belief model: model diagnosis

We carried out model diagnosis analyses to evaluate the goodness-of-fit of the DBM with the behavioral data. The model diagnosis analyses indicated good model fit of the DBM. See Supplementary Method and Supplementary Results for details.

### Dual control measures predict core symptoms of ADHD

To examine whether behavioral measures of dual control mechanisms (i.e., SSRT, context, performance monitoring, and anticipation-triggered proactive control), can predict core symptoms of ADHD (inattention and hyperactivity/impulsivity), we conducted multiple linear regression analysis and evaluated the model performance using leave-one-out cross-validation (LOOCV). Each time, one data point was selected as a test set and the rest of the data were used as a training set. The training set was then used to train a multiple linear regression model, which was then applied to the test set for classification. This procedure was repeated *N* times with each data point used exactly once as a test set. *Pearson’s* correlations were used to evaluate the correspondence between predicted values and observed values.

To further examine whether proactive control components play an important role in predicting core symptoms of ADHD, we trained a multiple linear regression model based on a reactive control measure alone, i.e., SSRT, and then compared model performance with the model trained on dual control measures.

## Supplementary information

Supplemental Material

## Data Availability

Code is available upon request.
