# Supplementary material for: Both reactive and proactive control are deficient in children with ADHD and predictive of clinical symptoms
Source: Transl Psychiatry. 2023 May 26;13:179. doi: 10.1038/s41398-023-02471-w (PMC10220086; doi:10.1038/s41398-023-02471-w)
Supplement: Supplementary file 1 — Supplemental Material [file 41398_2023_2471_MOESM1_ESM.pdf]

Supplementary Information

Supplementary Methods

Medication status

Among 50 participants with ADHD included in the final data analysis, 10 participants took Adderall, 8 took Concerta, 4 took Ritalin, 1 took Focalin, 1 took Metadata, 2 took Vyvanse, and 2 took Starterra. Twenty participants who were under stimulant treatment had gone through washout period of at least 5 half-lives of the medicine before testing.

ADHD Comorbidity

ADHD with conduct disorder (CD) and oppositional defiant disorder (ODD) were not excluded as they are common comorbidities [7, 8]. Total 16 CD and 24 ODD were confirmed with elevated t scores (>65) in CD and ODD scales in *Conners* 3rd Edition, respectively.

Drift Diffusion Model: model diagnosis

To assess the model's performance, we carried out a model diagnosis analysis. Initially, we utilized *fast-dm*[1] for the generation of behavioral data. The simulation was based on latent decision-making parameters specific to each condition (GoPSG and GoPUS) and subject. Each subject's simulation include 30 GoPSG and 30 GoPUS trials, which were repeated 100 times. As a result, there were 3000 simulated data for each set of subject-wise condition-specific latent decision-making parameters. Next, we conducted several statistical tests to assess the goodness-of-fit of the model with the behavioral data. First, we examined whether there was a significant post-error slowing in the simulated data by comparing RT in the GoPUS versus GoPSG trials. Second, we examined whether RT in the simulated data was highly correlated with the behavioral data. Last, we computed the root mean square error (RMSE) between the simulated and behavioral data and tested the significance using a permutation procedure. For each subject, we calculated RMSE of post-error slowing between the simulated and behavioral data. Then, we randomly shuffled the subject labels for simulated data and computed the permutated RMSE. We repeated the random shuffling step 500 times to generate a null distribution of RMSE, from which we calculated the p value of the RMSE from the behavioral data can be computed. **Supplementary Figure S5** illustrates the null distribution of the RMSE for post-error slowing.

Dataset for tuning DBM model parameter

We leveraged an independent dataset from a previous study [2] to optimize age-appropriate parameter for the DBM. The dataset included 38 subjects (12 female, all right handed with no history of neurological or psychiatric disorders, 9-12 years of age). Each child performed 2 runs of the SST. Subjects were instructed to respond as quickly as possible to green arrows (Go Signal) by clicking with their right pointer or middle finger based on the direction of the arrow. In 25% of the trials, after a variable delay, the green arrow turned red (Stop Signal), indicating that the subject should cancel their response. The delay between the Go Signal and the Stop Signal, the SSD varied across trials in a step-wise fashion and adjusted dynamically to the subject's performance: beginning at 165ms, it decreased by 33ms for a failed stop, and increased by 33ms for a successful stop.

Dynamic Belief Model: model diagnosis

The goodness fit of the DBM was evaluated by examining whether subjects actually adjust their response strategies based on model-estimated p_{stop}. The DBM predicts that subjects would slow down their responses when their expectation for stop signals increases. This prediction leads to two tests: (1) positive correlation between p_{stop} and RT on Go trials and (2) positive correlation between p_{stop} and Accuracy on Stop trials. The test was conducted on the pooled trial-wise data across all subjects.

Supplementary Results

No significant difference in cognitive performance between children with ADHD who have been treated with medication and those who do not

Children with ADHD who are not treated with medication and children with ADHD who have treated with medication did not show significant differences in any behavioral measure in this study, including Go accuracy, Go RT, Stop Accuracy, SSRT, post-error slowing and correlation between go RT and p_{stop} in the SST and Uncertain Go Accuracy, Uncertain Go RT, Certain Go Accuracy, Certain Go RT, Stop Accuracy, SSRT, and response slowing in the CSST (all *ps*>0.1)

No significant difference in cognitive performance between children with and without comorbidity

Children with ADHD with comorbidity of CD and/or ODD and children with ADHD without comorbidity of CD and/or ODD did not show significant differences in any behavioral measure in this study, including Go accuracy, Go RT, Stop Accuracy, SSRT, post-error slowing and correlation between go RT and p_{stop} in the SST and Uncertain Go Accuracy, Uncertain Go RT, Certain Go Accuracy, Certain Go RT, Stop Accuracy, SSRT, and response slowing in the CSST (all ps>0.2).

No significant difference in clinical symptoms between children who do not meet task performance criteria and children included in the data analysis

A total of 107 children aged 9-12 years were recruited from the local community for the study with 85 participants successfully completed all the required neuropsychological tests and two versions of the stop signal tasks. However, 5 participants were removed from the final data analysis due to their poor behavioral performance, which was determined by the criterion of Go accuracy less 50% and Stop accuracy out of the range from 25% to 75%. It is worth noting that of the 5 excluded participants, 3 were diagnosis with ADHD while the other 2 were not. The inattention and impulsivity/hyperactivity scores measured using the SWAN scale were not significantly different between the participants included in the final data analysis and those excluded due to poor behavioral performance (Inattention: t=0.66, p=0.5; Impulsivity/Hyperactivity: t=0.38, p=0.7).

Drift Diffusion Model: model diagnosis results

First, there was a significant post-error slowing effect in the simulated data that RT in GoPUS (545±100ms) is significantly longer than RT in GoPSG (515±86ms) (t=4.6, p=1.4e-05, paired t-test).

Second, there was a significant correlation in post-error slowing effect between the simulated data and behavioral data (r=0.88, p=2.2e-16, *Pearson's* correlation).

Third, the RMSE between simulated and behavioral data was significantly small (RMSE=59.84, $p<0.002$, permutation test).

In summary, model diagnosis analysis indicated good model fit of DDM in the post-error slowing effect.

Determining the optimal DBM parameter

To determine age-appropriate parameter for the DBM, we tested α ranging from 0.2 to 0.7 with incremental step of 0.05. For each α, we computed trial-wise p_{stop}, binned the data for each small range of p_{stop} in the aggregate trial-wise data across participants, computed average p_{stop} and α within each data bin, and calculated correlation between binned p_{stop} and binned go RT. The correlation coefficient increases when α changes from 0.2 to 0.3, it saturates at α of 0.3, and drops at α of 0.45 (**Supplementary Figure S3**). Therefore, we determined that 0.3 is the age-appropriate α value for our sample group. The p_{stop} is significantly correlated with go RT (r=0.57, p<0.001) and with Stop accuracy (r=0.61, p<0.001) at α=0.3 (**Supplementary Figure S4**).

Robustness of DBM results with respect to the choice of the model parameter

To examine whether our findings are dependent on the specific choice of the model parameter predetermined in the DBM, we conducted the same analyses as reported in the main text but used a different set of α ranging from 0.2 to 0.7 with incremental step of 0.1. Specifically, we examined whether we can replicate (1) significant difference in proactive control induced by signal anticipation between children with ADHD and TD children; (2) significant correlation between reactive control and proactive control induced by signal anticipation in TD children but not in children with ADHD; (3) significant correlation between clinical symptoms and proactive control induced by signal anticipation. It turns out that all these results are stable with varying α value (**Supplementary Table S3**).

Dynamic Belief Model: model diagnosis results

First, there was a significant positive correlation between p_{stop} and RT on Go trials (r=0.84, p=1.4e-11, *Pearson's* correlation, **Supplementary Figure S6a**).

Second, there was a significant positive correlation between p_{stop} and Accuracy on Stop trials (r=-0.48, p=0.002, *Pearson's* correlation, **Supplementary Figure S6b**).

This demonstrates that p_{stop} estimated from the DBM can predict subjects' adjustment of response strategy, indicating the goodness fit of the model.

Supplementary Figure S1. Reactive control in relation to ADHD core symptoms. The SSRT in the SST is significantly correlated with (**a**) inattention (*r*=-0.29, *p*=0.009) and (**b**) hyperactivity/impulsivity (*r*=-0.31, *p*=0.004) scores in SWAN. The relationship between reactive control and ADHD symptoms is replicated in the CSST, whose SSRT is significantly correlated with (**c**) inattention (*r*=-0.29, *p*=0.009) and (**d**) hyperactivity/impulsivity (*r*=-0.31, *p*=0.004) scores in SWAN.

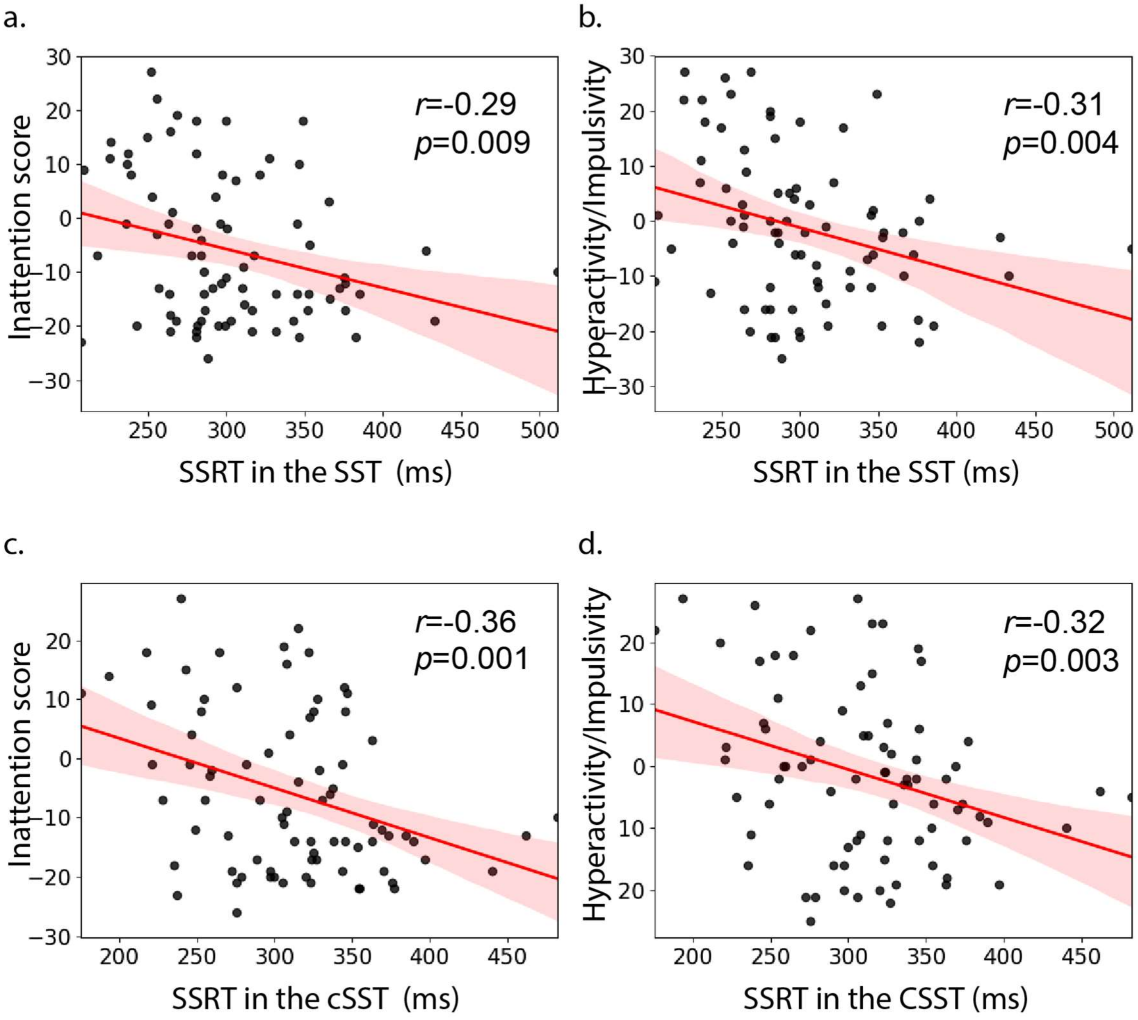

Supplementary Figure S2. Proactive control in relation to ADHD core symptoms. The negative feedback triggered proactive control, or post error slowing measured by RT difference between GoPUS and GoPSG is marginally significantly correlated with (**a**) inattention (*r*=0.2, *p*=0.06) and significantly correlated with (**b**) hyperactivity/impulsivity (*r*=0.33, *p*=0.003) scores in SWAN. The anticipation triggered proactive control measured by the correlation coefficient between trial-wise p_{stop} and RT is significantly correlated with (**c**) inattention (*r*=0.28, *p*=0.01) and (**d**) hyperactivity/impulsivity (*r*=0.31, *p*=0.004) scores in SWAN.

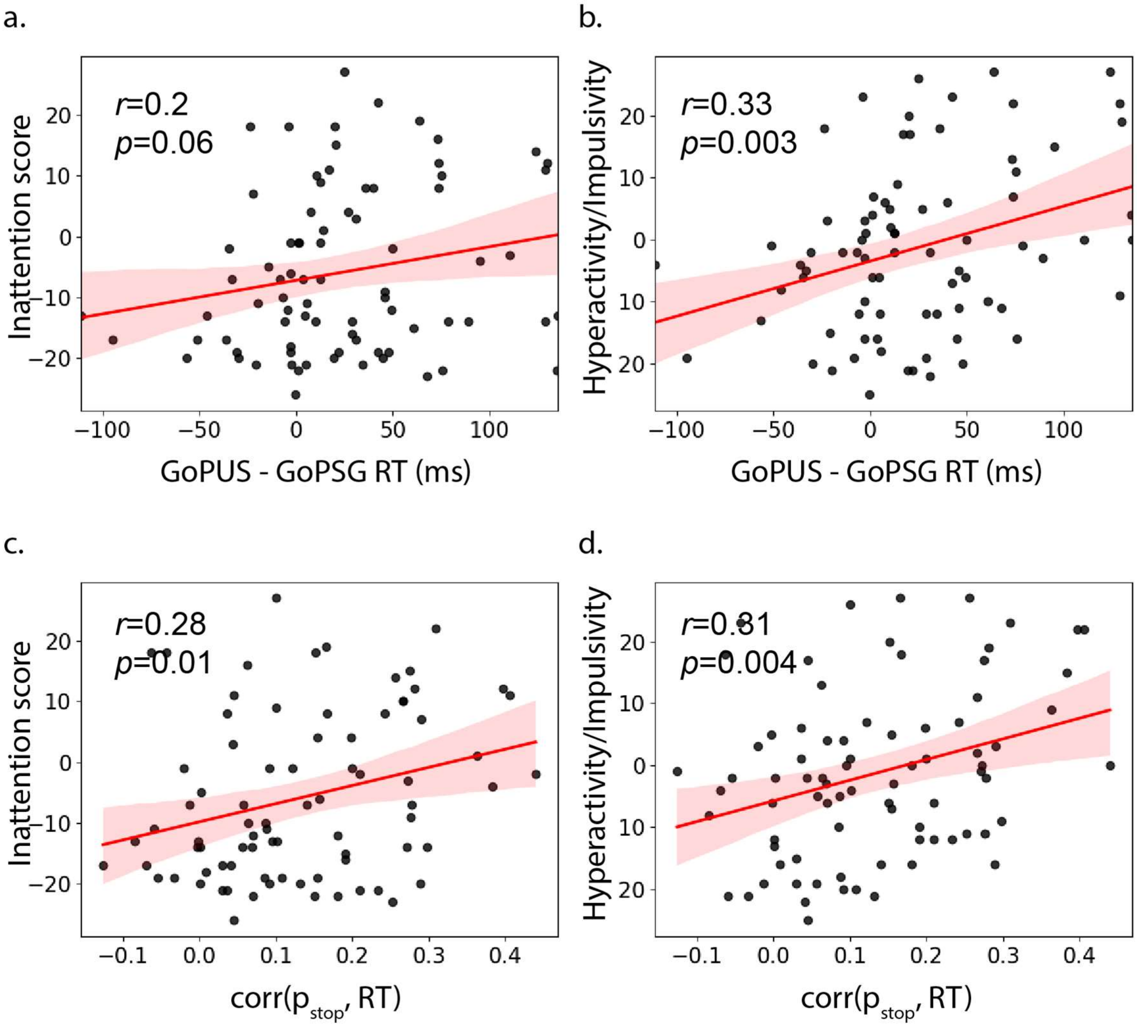

Supplementary Figure S3 Determine the optimal α value based on correlation coefficient between p_{stop} and go RT using an independent dataset involving young participants at age 9-12 years old.

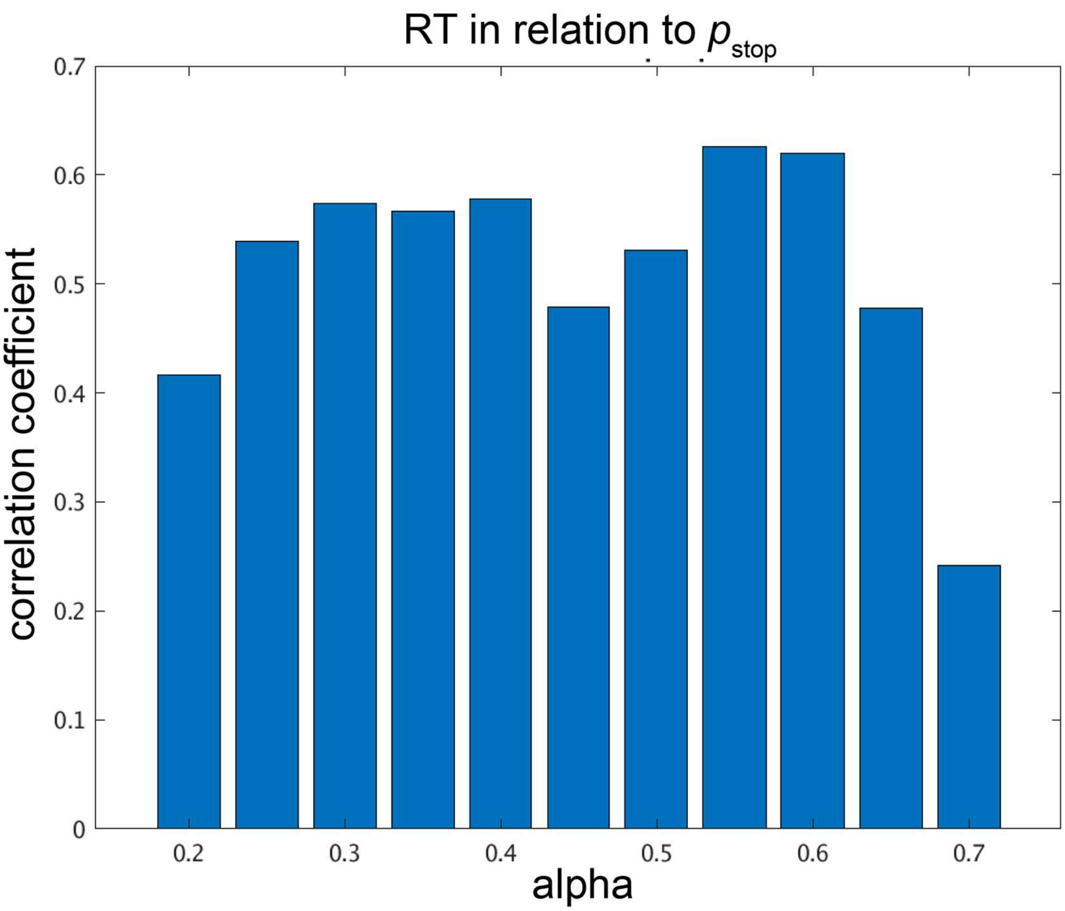

Supplementary Figure S4 The p_{stop} is significantly correlated with go RT (r=0.57, p<0.001) and with Stop accuracy (r=0.61, p<0.001) at α=0.3. This is demonstrated using an independent dataset involving young participants at age 9-12 years old.

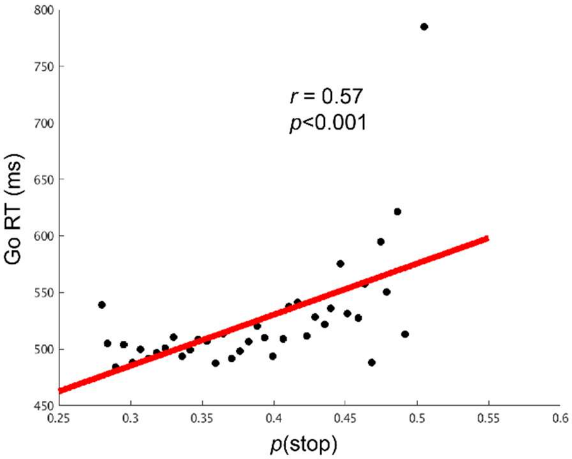

Supplementary Figure S5 Illustration of null distribution of RMSE. The goodness fit of the DDM estimation on GoPSG and GoPUS trials was evaluated using root mean square error (RMSE) between simulated and actual post-error slowing and its significance was tested again a null distribution of RMSE. The blue line shows the null distribution of RMSE, which was generated from 500 times permutations and red dot shows the RMSE between simulated and actual post-error slowing.

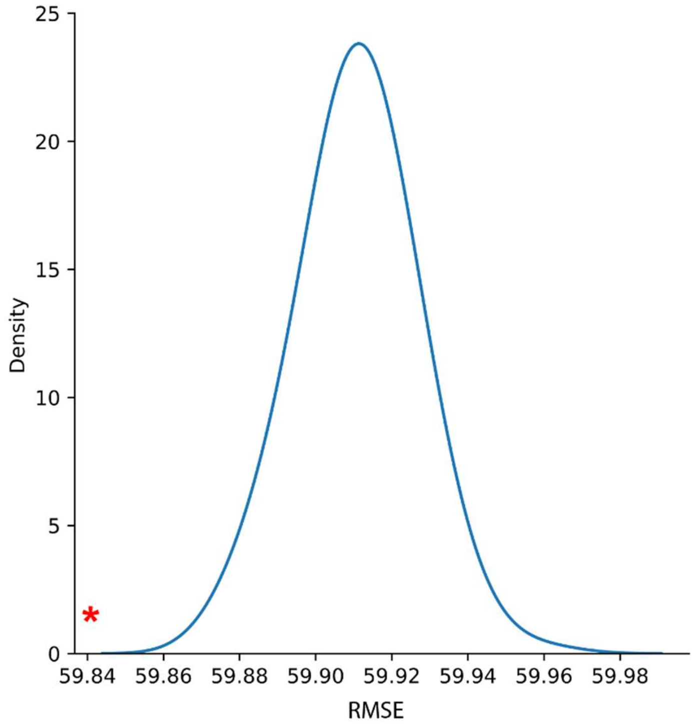

Supplementary Figure S6 Illustration of mode fitting performance of the DBM. The p_{stop} is significantly correlated with (**a**) go RT (*r*=0.84, *p*=1.4e-11) and (**b**) Stop accuracy (*r*=0.48, *p*<0.002).

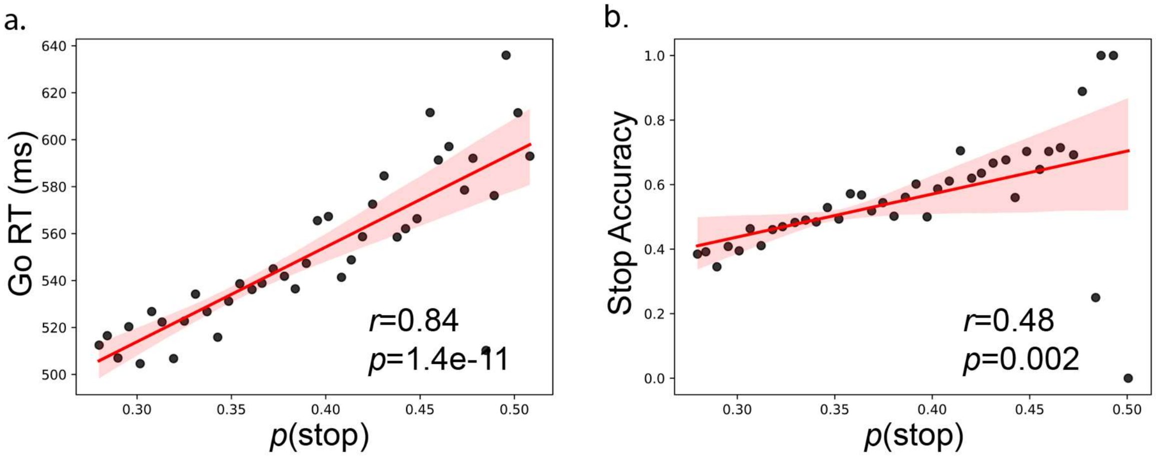

Supplementary Table S1. Multiple linear regression analysis revealed that proactive control measures is associated with reactive control measure (SSRT) in TD children after controlling potential confounds, including age, gender and verbal IQ.

	beta	t-value	p-value
Proactive control induced by task context			
Response slowing in CSST	-0.85	-2.37	0.03*
Age	-11.96	-1.68	0.1
Gender	-29.08	-1.74	0.09
Verbal IQ	0.49	0.69	0.5
Proactive control induced by performance monitoring			
PES in SST	-0.31	-2.02	0.05
Age	-16.91	-2.97	0.006
Gender	-16.24	-1.26	0.22
Verbal IQ	-0.25	-0.5	0.62
Proactive control induced by anticipation of stop signal			
Corr(p_{stop}, RT)	-94.88	-1.95	0.06
Age	-19.08	-3.49	0.002
Gender	-6.5	-0.5	0.62
Verbal IQ	-0.25	-0.5	0.62

Supplementary Table S2. Multiple linear regression analysis revealed that dual control measures are the robust predictors for the core symptoms of ADHD after controlling potential confounds, including age, gender and verbal IQ.

	Inattention				hyperactivity/impulsivity		
	beta	t	p		beta	t	p
Reactive control in relation to ADHD symptoms (SST)							
SSRT in SST	-0.07	-2.5	0.01*		-0.07	-2.5	0.01*
Age	-1	-0.73	0.47		0.04	0.03	0.97
Gender	-5.29	-1.77	0.08		-6.05	-2.03	0.046*
Verbal IQ	0.86	0.76	0.45		0.07	0.6	0.55
Reactive control in relation to ADHD symptoms (replication in CSST)							
SSRT in CSST	-0.97	-3.64	0.0005***		-0.08	-3.04	0.003**
Age	-1.23	-0.95	0.35		0.03	0.02	0.98
Gender	-6.84	-2.37	0.02*		-7.44	-2.53	0.01*
Verbal IQ	0.06	0.54	0.59		0.05	0.48	0.63
Proactive control in relation to ADHD symptoms (post-error slowing)							
PES in SST	0.04	1.5	0.14		0.08	2.72	0.008**
Age	0.17	0.13	0.9		1.17	0.93	0.36
Gender	-5.18	-1.68	0.09		-5.54	-1.86	0.07
Verbal IQ	0.13	1.1	0.27		0.09	0.83	0.41
Proactive control in relation to ADHD symptoms (anticipation)							
Corr(p_{stop}, RT)	30.4	2.46	0.01*		35.69	2.95	0.004**
Age	0.26	0.2	0.84		1.31	1.04	0.3
Gender	-6.16	-2.06	0.04*		-7.01	-2.39	0.02*
Verbal IQ	0.06	0.49	0.62		0.02	0.2	0.84

Supplementary Table S3 Robustness of main findings with respect to the choice of α value in the DBM (optimized value is 0.3 based on independent dataset).

	α value					
	0.2	**0.3**	0.4	0.5	0.6	0.7
Group difference in proactive control induced by signal anticipation						
t	3.13	3.24	3.07	3	2.9	2.81
p	0.003	0.002	0.003	0.004	0.005	0.007
Correlation between reactive control and proactive control induced by signal anticipation in TD children						
r	-0.38	-0.38	-0.44	-0.47	-0.5	-0.53
p	0.04	0.04	0.02	0.009	0.005	0.002
Correlation between reactive control and proactive control induced by signal anticipation in children with ADHD						
r	-0.01	-0.02	0.08	-0.11	-0.14	-0.15
p	0.95	0.85	0.57	0.44	0.34	0.28
Correlation between inattention symptoms and proactive control induced by signal anticipation						
r	0.3	0.28	0.27	0.26	0.25	0.24
p	0.007	0.01	0.01	0.02	0.02	0.03
Correlation between impulsivity symptoms and proactive control induced by signal anticipation						
r	0.33	0.31	0.32	0.31	0.31	0.3
p	0.002	0.004	0.004	0.005	0.006	0.006

Reference

1. Voss A, Voss J. Fast-dm: a free program for efficient diffusion model analysis. *Behav Res Methods* **39**, 767-775 (2007).

2. Cai W*, et al.* Hyperdirect insula-basal-ganglia pathway and adult-like maturity of global brain responses predict inhibitory control in children. *Nat Commun* **10**, 4798 (2019).
